# Effects of a Mindfulness-Based Intervention on Distress, Weight Gain, and Glucose Control for Pregnant Low-Income Women: A Quasi-Experimental Trial Using the ORBIT Model

**DOI:** 10.1007/s12529-019-09779-2

**Published:** 2019-04-16

**Authors:** E. Epel, B. Laraia, K. Coleman-Phox, C. Leung, C. Vieten, L. Mellin, J. L. Kristeller, M. Thomas, N. Stotland, N. Bush, R. H. Lustig, M. Dallman, F. M. Hecht, N. Adler

**Affiliations:** 1 Center for Health and Community, University of California, San Francisco, 3333 California St, San Francisco, CA 94143 USA; 2 School of Public Health, University of California, Berkeley, 50 University Hall #7360, Berkeley, CA 94720 USA; 3 Department of Nutritional Sciences, School of Public Health, University of Michigan, 1415 Washington Heights, SPH I 3866, Ann Arbor, MI 48104 USA; 4 Department of Psychology, Indiana State University, 200 North Seventh St, Terre Haute, IN 47809 USA; 5 Osher Center for Integrative Medicine, 1545 Divisadero St, San Francisco, CA 94115 USA

**Keywords:** Pregnancy, Mindfulness, Stress, Insulin resistance, Depression, Gestational weight gain

## Abstract

**Background:**

Stress can lead to excessive weight gain. Mindfulness-based stress reduction that incorporates mindful eating shows promise for reducing stress, overeating, and improving glucose control. No interventions have tested mindfulness training with a focus on healthy eating and weight gain during pregnancy, a period of common excessive weight gain. Here, we test the effectiveness of such an intervention, the Mindful Moms Training (MMT), on perceived stress, eating behaviors, and gestational weight gain in a high-risk sample of low income women with overweight/obesity.

**Method:**

We conducted a quasi-experimental study assigning 115 pregnant women to MMT for 8 weeks and comparing them to 105 sociodemographically and weight equivalent pregnant women receiving treatment as usual. Our main outcomes included weight gain (primary outcome), perceived stress, and depression.

**Results:**

Women in MMT showed significant reductions in perceived stress (*β* = − 0.16) and depressive symptoms (*β* = − 0.21) compared to the treatment as usual (TAU) control group. Consistent with national norms, the majority of women (68%) gained excessive weight according to Institute of Medicine weight-gain categories, regardless of group. Slightly more women in the MMT group gained below the recommendation. Among secondary outcomes, women in MMT reported increased physical activity (*β* = 0.26) and had lower glucose post-oral glucose tolerance test (*β* = − 0.23), being 66% less likely to have impaired glucose tolerance, compared to the TAU group.

**Conclusion:**

A short-term intervention led to significant improvements in stress, and showed promise for preventing glucose intolerance. However, the majority of women gained excessive weight. A longer more intensive intervention may be needed for this high-risk population.

Clinical Trials.gov #NCT01307683.

## Introduction

Pregnancy is a critical period for both maternal and infant health. Almost half of women begin pregnancy either overweight or obese, and a similar proportion gain excessive weight across their pregnancies [1, 2]. Women who begin pregnancy overweight or obese are more likely to gain in excess of the 2009 Institute of Medicine (IOM) guidelines compared to normal and underweight women [2, 3]. Excess gestational weight gain is a risk factor for numerous obstetrical complications, such as gestational diabetes [4], pregnancy-induced hypertension and toxemia [5, 6], Cesarean section delivery [5], and postpartum weight retention [7]. Excessive gestational weight gain in women with obesity is associated with offspring with macrosomia [5, 6, 8], hypoglycemia [9], preterm birth [8], childhood obesity, and metabolic syndrome [10–12].

In response to recognition of the importance of avoiding excessive weight gain during pregnancy, over 50 interventions to reduce gestational weight gain have been developed in the last decade [13]. Intensive programs addressing diet and/or exercise have shown some success in limiting weight gain [14]. However, there is tremendous heterogeneity of findings across studies. A recent, large systematic review found that among overweight and obese pregnant women, diet and exercise interventions vary widely in their effectiveness at limiting excessive gestational weight gain [13]. Some of this variation is likely due to differences in participant socioeconomic status and obesity, both risk factors for excessive weight gain.

Despite greater prevalence of excessive weight and obesity among African-American and Hispanic women, we know of only four gestational weight gain interventions that have been developed specifically for minorities—for African-American women [15, 16] and racially and ethnically diverse populations [17, 18]. Two of these interventions demonstrated successful reductions in mean gestational weight gain and in the proportion of women exceeding IOM guidelines [16, 17]. While promising, these pilot studies were small, with less than 35 participants in each group. Furthermore, none of the intervention studies reviewed included stress reduction as a way to improve metabolic health.

Chronic stressors such as work stress can lead to either weight gain or weight loss in animal studies and prospective studies in humans [19, 20]. However, regardless of direction of weight change, chronic stressors often lead to worsening of metabolic control, such as impaired glucose or insulin resistance [21], and increase the reward value of highly palatable food [22, 23]. There are sparse but suggestive studies during pregnancy showing higher levels of psychological distress—stress, anxiety, and depression—are associated with greater gestational weight gain [24, 25]. Greater psychological distress during pregnancy is associated with higher fasting glucose, apparently mediated by greater body mass index (BMI) [26]. In one study of over 800 pregnant women, having the combination of healthy diet, increased activity, and lower levels of distress appeared protective against glucose intolerance [27].

Here, we focus on a sample of low-income women, predominantly identifying as racial and ethnic minorities. We targeted low-income women because they are the highest risk group for stress and weight gain—they are exposed to greater financial and other stressors, and they are more likely to be obese, which in turn predicts excessive weight gain, worse maternal health and birth outcomes [28]. By restricting to low income, we also reduce the structural confounding of race and income that often comes with including a range of income, where minorities are overrepresented at the low end of income [29].

Mindfulness-based interventions have the potential to improve pregnancy stress and metabolic health. A meta-analysis of studies on non-pregnant adults found that mindfulness-based stress reduction can reduce perceptions of anxiety and depression [30]. Studies of mindfulness based stress reduction during pregnancy have also been done, with some finding reductions in depression and anxiety [31–33]. Mindfulness-based interventions targeting eating behavior can reduce dysregulated eating, including binge eating [34–37], improve glucose control [38], and in some studies promote weight loss in general samples with obesity [39]. Therefore, practice of mindfulness in daily life situations, and when applied to eating, could temper the rapid weight gain typical during pregnancy. The current study reports on the outcomes of a newly developed mindfulness based intervention designed to target psychological distress and overeating during pregnancy.

Given the limited success of weight loss interventions, a group of NIH leaders and researchers created the Obesity-Related Behavioral Intervention Trials (ORBIT) model for behavioral treatment development [40]. Developing interventions for eating behavior that work on novel mechanisms requires a flexible process, with clinically meaningful milestones. This model starts with a theory grounded in behavioral science about the mechanisms of behavior change, and develops an intervention in small incremental steps, paying attention to tailoring, acceptability, and testing the intervention’s efficacy of early outcomes using smaller experiments, before embarking on a randomized controlled trial.

We first conducted focus groups with the target population, which revealed high levels of interest in learning stress reduction techniques and acceptableness of mindfulness techniques [41]. Based on the science of stress and eating, we designed an 8-week, novel mindfulness-based stress reduction and healthy eating intervention by integrating critical components of prior mindfulness-based intervention studies focusing on pregnancy, mindful eating, and stress reduction [35, 36, 38, 41]. Further, a small proof-of-concept study found the intervention was acceptable, decreased distress, and increased acceptance of negative states [42]. We then further developed and optimized the intervention, described in detail elsewhere [43].

**Fig. 1:**
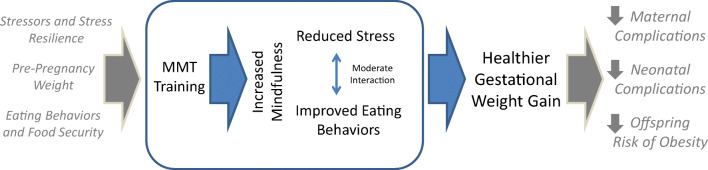
Theoretical model (reprinted from Vieten et al., [43])

Following the ORBIT model of intervention development, this study focuses on testing part of our theoretical model. The focus at this stage of the research was to tailor an intervention to meet the needs of the specific population, and to examine mechanisms and efficacy of early outcomes. Our model in Fig. 1 below, as detailed in Vieten et al. [43], proposes that changes in aspects of mindful attention and mindful eating (proximal mechanisms, treatment targets) will lead to decreased stress and improved behavioral risk factors (eating behavior). As described in Vieten et al., in examining the intervention group, we found initial evidence of the purported mechanisms in that the presumed proximal mechanism, mindfulness, did increase in the intervention group, and those increases were associated with decreases in distress and reductions in dysregulated eating behavior over the same time period [43]. The current study moves to the next step of the ORBIT model [40], testing efficacy on our biomedical outcome of weight gain.

Here, we tested the efficacy without a randomized control group, but rather with a comparison group receiving treatment as usual. We hypothesized that the intervention would significantly reduce the number of women gaining excessive weight (primary outcome) and decrease levels of perceived stress, depression, and poor metabolic health (based on glucose levels after an oral glucose tolerance test (OGTT)) (secondary outcomes).

## Methods

All procedures were approved by the University of California San Francisco, California Pacific Medical Center, University of California Berkeley, and Contra Costa Regional Medical Center and Health Centers Institutional Review Boards.

### Study Population

Our inclusion criteria included English-speaking women with singleton pregnancies, aged 18–45, with a self-reported pre-pregnancy BMI between 25 and 41 kg/m^2^, and with a household income less than 500% of the federal poverty level.^1^ Women had to be 12–19 weeks’ gestation at the start of the intervention, and those in the intervention had to be able to attend 8 weekly two hour intervention classes. We limited gestational age to ensure that women were past their first trimester, when risk of miscarriage is high, and early enough in pregnancy for the intervention to still impact gestational weight gain. All women enrolled had to be from 19 to 45 years, able to complete forms in English, not have a substance abuse, mental health, or medical condition that, in the opinion of investigators, would make it difficult for the potential participant to participate in a group intervention. Exclusion criteria included the inability to complete forms in English, needle phobia or fainting response, substance abuse, medical conditions that might affect gestational weight gain (including known diabetes, HIV, hypertension, and eating disorders), polycystic ovarian syndrome treated with metformin, a regular meditation practice (20 or more minutes two times or more a week), recent weight loss (more than 5% within 6 months), chronic use of corticosteroids, or a history of gastric bypass surgery. We did not exclude women who have had previous children.

### Recruitment

We first conducted a pilot trial to determine feasibility and to refine recruitment strategies [44]. For the current study, we recruited women from hospital-based clinics, community health centers, Supplemental Nutrition Assistance Program (SNAP) and Women, Infants, and Children (WIC) offices, organizations providing services to pregnant women, and through online advertisements (e.g., Craigslist) from August 2011 to June 2013 with the goal of enrolling 220 women. Women were recruited and enrolled in sequential “waves” to form groups of 8–12 women with expected dates of delivery within 2 months of each other. Details of our recruitment strategy have been published previously [44]. We included an orientation session where we described the pros and cons of being in a study, and the importance of adherence to the study, to reduce dropouts [45].

#### Non-Randomized Control Group (Treatment as Usual or TAU)

While it is always preferable to have a randomized control group, this is less critical in the early stages of intervention development. The current study was an initial efficacy study where we were piloting a new intervention. As described in the “Introduction” section above, based on the ORBIT model, studies testing the development of a new intervention are encouraged to use a socio-demographically similar comparison group. Because our group class format required enrolling women at the same stage of pregnancy, it was difficult to recruit sufficient numbers for a wave, and it would have been problematic to use only half for the treatment group, given the desired group class size of 10 women. Therefore, women unable to attend intervention classes due to their schedules, or women with gestational age of 20–23 weeks who otherwise met the eligibility criteria, were eligible for being part of the “Treatment As Usual” (TAU) group**.** The treatment as usual included whatever prenatal medical care the individuals received on their own. Therefore, both the treatment group and the TAU group received medical care as usual, with the only difference being that the treatment group received the MMT classes as well. Due to this recruitment design, the TAU group had slightly later gestational age than the intervention group, a limitation addressed in the discussion.

### Study Design

Participants were asked to complete study questionnaires regarding psychological distress, eating behavior, and exercise at baseline and 8 weeks later (post-intervention). Participants were paid $25 for completing the measurement battery. Participants in the intervention were additionally compensated $25 for attending each session to help cover their time, transportation, and any childcare. After delivery, medical records were reviewed by trained research assistants to confirm pre-pregnancy BMI, when possible, and gestational age, assess total gestational weight gain based on pre-pregnancy BMI, code medical complications, and assess which participants may have developed gestational diabetes (based on glucose levels from the OGTT). Women in the MMT group came in person for their baseline assessment, but TAU did not.

### Intervention

The intervention development process was based on the ORBIT model [40]. This model guides early stages of intervention development, applying basic behavioral mechanisms of eating and optimizing delivery and dose before moving on to further stages of more formal testing. After completing a pilot study to confirm that the intervention we developed, the MAMAS (Maternal Adiposity, Metabolism, and Stress) Mindful Training, was affecting the purported mechanisms (stress and stress eating), we moved to this efficacy trial. The final intervention, the Mindful Moms Training (MMT), included 8 weekly 2-h sessions, two “booster” telephone sessions, and one postpartum group session with mothers and babies. The intervention was led by two practitioners who worked in pairs. They had graduate degrees (MA, certified nurse midwife, and PhDs), with additional training in both mindfulness and Mindfulness-Based Eating Awareness Training (MB-EAT) [36].

For sessions that included both experiential and didactic components integrated material from three empirically supported interventions: Mindful Motherhood [41], Mindfulness-Based Stress Reduction [46], and MB-EAT [36]. The sessions focused on three commitments, represented by the slogan “Mindful Eating, Move My Body, Breathe!” Participants were given a list of physical activities safe for pregnancy, which focused on ways to increase amount of daily walking and stretching.

Classes began with mindful movement and a check-in where each person shared their experiences with mindfulness practices in the past week. Didactic discussions then covered (1) stress reduction, focused on acceptance-based coping, awareness of breath, body, thoughts, and emotions; (2) mindful eating, focused on heightening awareness of hunger, fullness, taste experience, and thoughts and emotions leading to reactive and/or automatic eating; and (3) nutrition, focused on optimal foods to eat more of (e.g., whole foods), what to eat less of (e.g., processed foods), reading labels, identifying healthy portion sizes, and introducing both the plate method and food pyramid as resources. Each class ended with a minute mindfulness practice and a review of homework for the upcoming week. The curriculum model, development and content, and the high level of fidelity and adherence is described in detail in Vieten et al. [43].

### Measures

#### Weight, Metabolic Health, and Physical Activity

##### Gestational Weight Gain

The primary outcome was gestational weight gain category, based on the 2009 IOM recommendations. Total gestational weight gain was calculated as the difference between weight at the last prenatal visit before delivery, and self-reported pre-pregnancy weight that had been recorded in the medical record. If the last recorded prenatal weight occurred more than 30 days before delivery, total gestational weight gain was coded as missing (*n* = 23). When pre-pregnancy weight was not recorded in the prenatal record (*n* = 69), we used self-reported pre-pregnancy weight from the study eligibility screener.

### Secondary Outcomes

#### Six-Month Postpartum Weight Retention

This secondary outcome was calculated as the difference between the participant’s measured 6-month postpartum weight and their pre-pregnancy weight. For analysis, this was assessed both as a continuous variable and categorical: the difference in weight at 6 months was dichotomized as higher than their pre-pregnancy weight vs. equal to or less than their pre-pregnancy weight.

#### Psychosocial Distress, Mindfulness Outcomes, and Eating Behavior

The primary psychosocial outcomes were three standardized distress measures, with Cronbach’s *α* reliability for this sample as stated: (1) *global perceived stress*, using Cohen’s Perceived Stress Scale (*α =* 0.87), a 10-item measure of stress perceptions, including ratings of feeling overwhelmed, out of control, and stressed [47]; (2) *depressive symptoms*, using the Patient Health Questionnaire (PHQ-9) (*α* = 0.84), a 9-item scale of depressive symptoms used in primary care settings [48, 49]; and (3) *pregnancy-related anxiety*, using the Pregnancy-Related Anxiety Scale (*α* = 0.87), a 10-item scale, that assesses the extent to which pregnant women are concerned about their health, their baby’s health, labor and delivery, and caring for their baby [50].

Secondary measures included the *acceptance of negative experiences* using the Acceptance and Action Questionnaire-II (*α* = 0.85). This measures the extent to which people are bothered by having negative thoughts and feelings, versus being able to accept them [51]. The eating behaviors measured included *emotional eating* (*α* = 0.96) and *external eating behavior* (α = .84) using the Dutch Eating Behavior Questionnaire [52], and level of *food addiction*, using the Yale Food Addiction Scale (*α* = 0.80) [53]. We also measured *trait mindfulness* in the intervention group, as reported elsewhere [54].

##### Oral Glucose Tolerance Test (OGTT)

As part of their usual prenatal care, 141 study participants (141/180 = 78% of final sample) completed an oral glucose tolerance test between 24 and 28 weeks’ gestation. Glucose levels from after the 1-h test were abstracted from the prenatal medical record. Level of impaired glucose tolerance was assessed using continuous values of glucose and impaired tolerance was categorically defined as a glucose level above 130 mg/dL. Comparison of OGTT cases to those without an OGTT showed no significant differences on BMI nor any of the demographic variables listed in Table 1.

**Table 1:** Baseline characteristics

	**Control (*n*** **= 105)**	**MMT (*n*** **= 110)**	***p*** **value**
	***n*** **(%)**	***n*** **(%)**	
Age (years), mean (SD)	28.0 (6.0)	27.8 (5.7)	0.88
Race/ethnicity, *n* (%)			0.65
White	15 (14.3)	14 (12.8)	
African American	45 (42.9)	39 (35.8)	
Latino	29 (27.6)	35 (32.1)	
Other/multiracial	16 (15.2)	21 (19.2)	
Education			0.07
<12 years	16 (15.2)	10 (9.1)	
High school graduate/GED	18 (17.1)	30 (27.3)	
Any college or vocational training	48 (45.7)	56 (50.9)	
College graduate or higher	23 (21.9)	14 (12.7)	
Marital status			0.76
Married or in committed relationship	72 (69.2)	74 (67.3)	
Single, separated, or divorced	32 (30.8)	36 (32.7)	
Household income, mean (SD)	$23,676 ($20,857)	$24,723 ($22,459)	0.74
Number of previous children, mean (SD)	1.0 (1.3)	0.8 (1.0)	0.14
Proportion with previous birth, mean (SD)	0.53 (0.5)	0.50 (0.5)	0.63
Pre-pregnancy weight status			0.28
Normal or overweight	53 (51.0)	58 (55.2)	
Class I obese	25 (24.0)	30 (28.6)	
Class II obese	26 (25.0)	17 (16.2)	
Food insecure	43 (41.4)	44 (41.9)	0.93
Smoking status			0.28
Current smoker	9 (8.7)	5 (4.8)	
Former smoker	50 (48.1)	44 (42.3)	
Never smoker	45 (43.3)	55 (52.9)	
Leisure-time physical activity			0.76
Inactive or light activity	59 (56.7)	58 (56.9)	
Moderate or vigorously around 3 times/week	25 (24.0)	21 (20.6)	
Moderate or vigorously on most days	20 (19.2)	23 (22.6)	
			0.55

##### Physical Activity

This was assessed at baseline and at post-intervention using the Stanford Brief Activity Survey, which provides a brief assessment of the amount and intensity of activity completed during leisure time [45]. Level of activity was reported as six levels, ranging from 1 (inactive) to 6 (very active). We used both the continuous score as well as a categorical analysis. The responses were further collapsed into three mutually exclusive categories: lowest level = inactive or light activity, medium level = moderate or vigorous activity 3 days per week, and highest level = moderate or vigorous activity at least 5 days per week.

### **Sociodemographics and Covariate**s

A parsimonious set of covariates were selected a priori for the two physiological outcomes (weight and glucose control) that are well-established correlates of gestational weight gain. Covariates included age at enrollment (continuous), parity (continuous), gestational age because weight gain is a function of the length of gestation, and pre-pregnancy BMI category (<30 kg/m^2^, 30.0–34.9 kg/m^2^, and ≥ 35.0 kg/m^2^). We used pre-pregnancy BMI category as a covariate rather than actual BMI because the IOM weight gain guidelines are based on pre-pregnancy BMI category, and the relationship between pre-pregnancy BMI and weight gain is non-linear.

We measured additional sociodemographic variables to further describe the sample. This included race and ethnicity (categorized as Caucasian, African American, Latino, and other/multiracial), educational attainment (<12th grade, high school graduate, any college/vocational training, or college graduate or higher), marital status (single versus married or in a committed relationship), household income (reported value), and smoking status (current, former, or never smoked). Household food security was measured with the 10-item U.S. Adult Food Security Module [55], which includes questions about limiting food intake or not eating balanced meals due to lack of money. Food insecure households were defined as answering “yes” to three or more questions. These additional sociodemographic variables are shown in Table 1; since they did not vary by group, they were not used as covariates.

### Statistical Analyses

All statistical analyses were performed using SAS version 9.3 (SAS Institute Inc., Cary, NC). Statistical significance was determined at *p* < 0*.*05, two-tailed. The analysis plan followed an intent-to-treat principle by comparing participants’ outcomes in the intervention (regardless of how many classes they attended) to outcomes of all participants in the TAU group. However, an as-treated analysis, comparing women who attended a minimum of five of the eight classes to women in the TAU group, yielded highly similar results. To deal with missing data, for gestational weight gain, analyses were performed on available data, where we removed cases in the presence of missing/incomplete data. For the self-report scales, for missing items, mean substitution was used, unless > 30% of scale items were missing.

Given that in our theoretical model (Fig. 1), changes in distress precede and will lead to changes in eating and weight, we first report the psychological variables, then weight change, our primary outcome, and lastly the other physical health outcomes. To examine the intervention effect on outcomes of psychosocial distress, eating behaviors, and mindfulness within each group, we first used paired *t* tests to compare baseline to post-intervention scores on our measurement scales. For our primary analysis, to test whether the change scores were significantly different when compared to the TAU group, multivariate linear regression models were used to compare the between-group changes, further adjusting for age and pre-pregnancy BMI.

To examine the intervention effect on gestational weight gain, a multinomial logistic regression model was fit for IOM gestational weight gain category, using “normal” weight gain (e.g., within the IOM guidelines based on a woman’s pre-pregnancy BMI) as the referent group. A sensitivity analysis that excluded women who developed gestational diabetes mellitus (GDM) after enrolling in the study was also conducted (*n* = 23). Covariates included age, pre-pregnancy BMI, and parity.

For secondary analyses, a multinomial logistic regression model was fit for post-intervention physical activity categories, adjusted for age, pre-pregnancy BMI, and parity. Multivariate logistic regression models were fit for outcomes of impaired glucose tolerance and 6-month postpartum weight retention, adjusted for the study covariates.

We examined the statistical power for this size of a sample. For a group means comparison, with our actual sample size of roughly 105 per group, we had 80% power to detect a small medium effect size (around *d* = 0.39). Another way of considering power for our primary analysis is that, given empirical data on similar samples [28], we expected around 60% to gain excessive weight, in which case we would have 80% power to detect an additional 20% of the MMT sample not gaining excessive weight compared to TAU group (so only 40% total with excessive weight gain), associated with MMT with regard to excessive weight gain, equivalent to an odds ratio of up to − 0.44 for excessive weight gain and a Cohen’s d of small to medium effect.

## Results

### Sample Descriptives and Consort Diagram

**Fig. 2:**
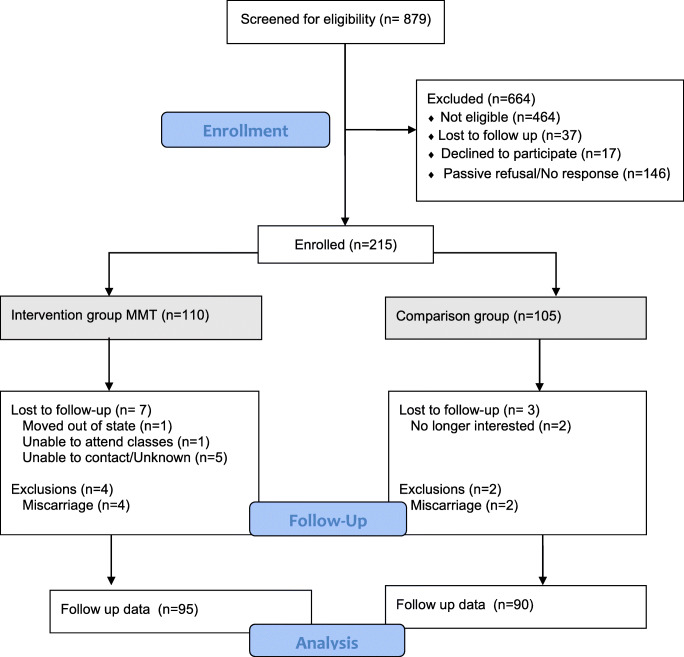
Screening, enrollment, and follow-up of study sample

Our recruitment and participant flow is shown in the consort diagram, Fig. 2. Of the 879 women we screened, 415 (47.2%) were eligible to participate in the study; 110 were enrolled in the intervention and 105 were enrolled in the TAU group (215 total). The most frequent reasons for ineligibility were BMI outside of the target range (*n* = 251), early or late gestational age (*n* = 58), medical or psychosocial reasons (n = 58), and high income (*n* = 46). During the intervention period, six participants had pregnancy losses (leaving 209 total participants). Ten participants were lost to the study (5% of enrolled sample): one participant moved out of state, one was unable to attend classes due to logistical reasons, and eight were lost to follow-up for unknown reasons. Our final sample included 95 intervention and 90 TAU participants who had weight outcomes (185/ 209), 89% of the remaining eligible sample. Due to missing data, there were fewer who completed the questionnaires at both timepoints: 82 intervention and 89 TAU participants (171/209, 82%). We included analyses on all data available, rather than excluding women without complete data.

Adherence to the intervention was high; 75% of women attended five or more of the eight group sessions. Results were also tested with “as-treated” group, and results did not change. Therefore, all results presented are with the intent-to-treat full sample. Baseline characteristics of the sample are shown in Table 1. The mean age of the sample was 27.9 years. Approximately 39% had a high school education or less, the average annual household income was less than $25,000, and 42% of women reported food insecurity. Most women (67%) were married or in a committed relationship. Women were screened and enrolled into the study using self-report of weight and height. According to their pre-pregnancy BMI based on the prenatal medical record, 4% of women were categorized as normal weight, 54% of women were overweight, and 47% were obese (26% with class 1 obesity and 21% with class 2 obesity). Eligibility was based on their initial self-reported pre-pregnancy BMI at screener (between 25 and 41 kg/m^2^) and thus we still included the women with BMI < 25. Their actual measured BMI for *n* = 9 was on average 24. (Note we later conducted a sensitivity analysis on our primary outcome—weight gain category—excluding these nine participants, and found that the results were similar.)

There were no significant differences in sociodemographic or health-related baseline characteristics between women in the intervention group versus the TAU group. For example, they had similar income levels, and had on average one child. We also tested whether the proportion of women who were primagravida/mulitiparous differed by group. There was a similar percentage in each group who were multiparous, with around 50% having already had a child (*χ*^2^ = 0.24, *p* = 0.63).

### Intervention Effects on Distress and Mindfulness

First, we examined within-group effects using paired *t* tests. The effects of the intervention on distress, acceptance of negative experiences, and eating behaviors are shown in Table 2. Overall, the intervention group showed significant decreases from baseline to the post-intervention period in distress (perceived stress and depression) and in eating-related behaviors (food addiction, emotional eating, external eating). There were also improvements in acceptance of negative experiences. With the exception of improvement in food addiction, there were no statistically significant improvements on any measures in TAU participants.

**Table 2:** Intervention effects on changes in psychosocial distress, mindfulness, and eating behaviors

	**Scale range**	**Control**		**MMT**		**Intervention effect**^**1**^		
		**Baseline (*n*** **= 104)**	**Post-intervention (*n*** **= 89)**	**Baseline (*n*** **= 106)**	**Post-intervention (*n*** **= 82)**			
		**Mean (SD)**	**Mean (SD)**	**Mean (SD)**	**Mean (SD)**	**Change**	**95% CI**	*P* value
Psychological distress								
Perceived stress	0–40	18.4 (6.6)	17.0 (7.4)	19.1 (6.6)	15.6 (5.8)*	− 2.09	− 4.04, − 0.14	0.04
Depressive symptoms	0–27	6.8 (4.9)	6.1 (4.5)	7.6 (5.6)	4.5 (3.7)*	− 1.95	−3.35, −0.55	0.007
Pregnancy anxiety	1–4	2.1 (0.7)	2.0 (0.6)	2.1 (0.6)	2.0 (0.7)	0.01	− 0.17, 0.19	0.9
Acceptance								
Experiential avoidance	7–70	50.8 (10.8)	51.0 (10.7)	49.2 (10.7)	53.7 (8.8)*	3.96	1.18, 6.74	0.006
Eating behaviors								
Food addiction (YFAS)	0–7	2.0 (1.6)	1.7 (1.4)*	2.2 (1.6)	1.7 (1.1)*	− 0.11	− 0.55, 0.32	0.61
Emotional eating (DEBQ)	1–5	2.0 (0.9)	2.0 (0.8)	2.1 (0.8)	1.9 (0.7)*	− 0.12	− 0.32, 0.09	0.26
External eating (DEBQ)	1–5	2.8 (0.7)	2.8 (0.6)	2.84 (0.6)	2.77 (0.5)*	− 0.13	− 0.29, 0.02	0.09

The sample size varies based on missing data, ranging from 167 to 170 for final sample with complete data for each measure

*p < 0.05

^1^The intervention effect tests the differential difference in the change scores, from pre to post, in the MMT vs. TAU groups

As expected, perceived stress, depressive symptoms, and acceptance of negative experiences were strongly related, with *r*’s = 0.56 to 0.58, as were emotional and external eating (*r* = 0.70). All other psychological measures were only moderately correlated (from 0.13 to 0.35).

We tested whether there were significant differences in the degree of change between the two groups. After adjustment for age and pre-pregnancy BMI, the intervention was related to significantly greater improvements in measures of psychological distress—perceived stress (*β* = − 2.01, 95% CI − 3.93, − 0.09), depression (*β* = − 2.00, 95% CI − 3.39, − 0.62), and acceptance (*β* = 3.39, 95% CI 0.53, 6.26). Eating behavior and other improvements showed marginal or no significant differences compared to those of the TAU group.

### Intervention Effects on Weight

As shown in Table 3, the majority of women in the sample gained excessive weight during pregnancy (68% on average across groups), with no differences between treatment groups (67.4% in MMT and 68.9% in TAU). We first analyzed weight change as a continuous variable using kilograms of weight change. We conducted a regression with the same model as used in Table 4, using the covariates maternal age, pre-pregnancy BMI category, and parity. The MMT group gained 0.05 kg more than TAU on average, not a significant difference between groups (*p* = 0.98).

**Table 3:** Intervention effects on gestational weight gain

**Weight change**	**Control**	**MMT**	**Intervention effect**		
	***n*** **(%)**	***n*** **(%)**	**OR**	**95% CI**	*P* value
Below IOM recommendations	10 (11.1)	19 (20.2)	**3.56**	1.17, 10.87	0.026
Within IOM recommendations	18 (20.0)	12 (12.6)	Referent		
Above IOM recommendations	62 (68.9)	64 (67.4)	1.39	0.60, 3.22	0.44

OR adjusted for age, pre-pregnancy BMI, parity

**Table 4:** Intervention effects on glucose tolerance, weight retention, and physical activity

	**Control**	**MMT**	**Intervention effect**		
	**Mean (SD)**	**Mean (SD)**		**95% CI,**	***p***
OGTT glucose levels (mg/dL) (24 weeks) (*n* = 141)	111.8 (27.7)	100.3 (23.3)	***β*− 11.78**	− 20.55, − 3.02	**0.009**
Oral glucose tolerance test at 24–28 weeks’ gestation			**OR**		
Normal (≤ 130 mg/dL)	54 (79.4)	67 (91.8)	Referent		
Impaired (> 130 mg/dL)	14 (20.6)	6 (8.3)	**0.34**	0.12, 0.95	**0.04**
Postpartum weight retention at 6 months (kg)	6.0 (8.4)	4.2 (8.8)	***β*− 0.89**	− 3.42, 1.64	0.49
			**OR**		
Postpartum weight retention at 6 months (categorical)					
Higher than pre-pregnancy weight	50 (78.1)	56 (70.0)	Referent		
Equal to or lower than pre-pregnancy weight	14 (21.9)	24 (30.0)	0.55	0.25, 1.23	0.145
			***β***		
Leisure-time physical activity (post-intervention)	2.7 (1.1)	3.3 (1.1)	0.58	0.26, 0.91	**0.0005**
Physical activity category	*N* (%)	*N* (%)	**OR**		
Inactive or light activity	44 (49.4)	20 (25.0)	Referent		
Moderate or vigorously active (around 3 times/week)	25 (28.1)	28 (35.0)	**2.55**	1.13, 5.72	0.02
Moderate or vigorously active (5 or more times/week)	20 (22.5)	32 (40.0)	**3.79**	1.60, 8.97	0.003

*β* adjusted for age at enrollment, pre-pregnancy BMI, and parity, and for physical activity post-intervention, adjusted for baseline physical activity

As part of examining IOM categories, we examined the group who gained “less than recommended”—most had not gained any weight, some gained minimal weight. Among MMT, 20% of the sample gained less than the IOM recommendations, and among TAUs, this occurred for 11% of the sample, a significant difference (Table 3). There was a significant association between being in MMT (vs. TAU) with more frequent gestational weight gain below the IOM recommendations (OR 3.83, 95% CI 1.24, 11.83) compared to the referent group of gaining within the IOM recommendations. In other words, there were more women who gained too little, according to the recommendations, in the MMT group.

To test if weight changes may have occurred due to a medical condition, sensitivity analyses were performed and showed that this effect persisted after excluding women who developed gestational diabetes mellitus (*n* = 23). It is also possible that the excessive weight gain was in part already determined by the first 20 weeks of pregnancy and therefore could not be influenced by the intervention. Therefore, sensitivity analyses were conducted to eliminate the 17% (*n* = 25) of women who had already gained in excess of the IOM recommendations at the start of the intervention period, around 20 weeks, regardless of group assignment. This exclusion did not change results.

Lastly, we conducted post hoc Pearson correlations between changes in reported distress and our primary outcome, changes in weight. There were no significant relationships for either stress (*r* = − 0.05) or for depressive symptoms (*r* = 0.06) with weight change.

*Postpartum weight retention*. At 6 months post partum, both groups showed weight retention (6 kg for TAU, 4 kg for MMT), but there was wide variance and no statistically significant differences between groups (Table 4). When examined categorically, 30% of women in the intervention were either at or below their pre-pregnancy weight, compared to 22% of women in the TAU group. After adjustment for covariates, MMT showed lower 6-month postpartum weight retention, but this was not significant (OR 0.55, 95% CI 0.25, 1.23).

*Physical activity* among women in the intervention and TAU conditions are shown in Table 4. Women in the intervention group reported higher levels of leisure-time physical activity than women in the TAU group covarying baseline levels (*β* = 0.58). After adjustment for covariates and baseline physical activity, the intervention was associated with greater odds of having high levels of activity, around 3 times/week (OR 2.55, 95% 1.13, 5.72) and around 5 times/week (OR 3.79, 95% CI 1.60, 8.97; Table 4).

#### Glucose Tolerance

Women in the MMT group started classes at 18 to 20 weeks of pregnancy and received at least half of the intervention classes before the OGTT was performed, at 24–28 weeks’ gestation. Glucose levels were examined both as a continuous measure and as a categorical measure (of impaired glucose tolerance), 1 h after the glucose drink (Table 4). The MMT group had mean levels of 100 mg/dL, whereas the TAU group had significantly higher mean levels of 111 mg/dL (*p* < 0.03). When examined categorically, approximately 8% of women in the intervention and 21% of women in the TAU group had a glucose level above 130 mg/dL. After adjustment for age, pre-pregnancy BMI, and parity, the intervention was associated with significantly lower odds of exhibiting impaired glucose tolerance (OR 0.34, 95% CI 0.12, 0.97).

## Discussion

We developed and tested an 8-week mindfulness-based, stress reduction and healthy eating intervention for low-income overweight women during pregnancy—the Mindful MAMAS Training (MMT). Our sample was a young, low-income, ethnically diverse sample of pregnant women who had high baseline levels of stress, food insecurity, and a sedentary lifestyle. We found that although women in MMT did not gain less weight than women in the comparison group (our primary outcome), they did demonstrate significant improvements on secondary outcomes: psychological stress and depression, two important mental health outcomes, as well as glucose control on the OGTT and reported physical activity. They had on average around 2 point reductions in stress and depression, post-intervention. As reported elsewhere, they were less likely to have significant depressive symptoms up to 9 months later [57] and their infants had almost half as many medical visits in the following year as the infants from the comparison group [58].

However, despite the pattern of significant improvements, the intervention had no effect on the primary clinical outcome of gestational weight gain. If improvements in stress were maintained over time, it should theoretically temper the excessive weight gain. In this study, in post hoc exploratory analyses, we did assess relations between self-reported stress with weight change, but found no significant relationships. This lack of relationship may indicate that the model does not apply well to this sample, or, quite possibly, it could be due to the limited measurement design (measured only pre/post and using trait measures) and/or because the majority gained excessive weight, leaving little variance to explain in weight outcomes.

Other improvements seen, such as lower distress and glucose tolerance, may be equally important to maternal and offspring health as the level of weight gain. The better glucose control during pregnancy is important both for maternal and infant health. The finding that mindful eating training was associated with an improvement in glucose homeostasis, in the absence of differences in weight gain, in comparison to a TAU group, has been found in two other studies of non-pregnant adults—one study of mindful eating in men and women with obesity [39], and a randomized controlled trial comparing a mindful eating intervention and a diabetes self-management education intervention [59]. Given this preliminary pattern of findings on glucose but not weight across three studies now, it is possible that mindful eating has larger effects on glucose than on weight loss or maintenance but this requires further investigation.

The intervention taught mindful eating skills, thus it was not surprising that the MMT group reported significant improvements in measures of eating in response to external cues or emotions, and the change in external eating was marginally significantly greater compared to the TAU group. It is not known if this was due to demand characteristics or actual changes in eating behavior. Both groups improved on a measure of food addiction, an unexpected finding. While it is possible that reductions in strong drive to eat might occur naturally during pregnancy, this needs further study.

Despite the benefits in emotional well-being and glucose control, we failed to find a significant difference between intervention and control participants on our primary outcome of weight gain. Almost 70% of women in the sample, regardless of group, gained excessive weight. One reason may be that the sample studied here tends to be among the highest risk for excessive gestational weight gain, with the co-occurrence of several major risk factors—pre-pregnancy overweight or obese status, low income, food insecurity, and minority status [60–62]. In another overweight minority pregnant sample, a similarly high number, 60%, were reported to gain excessive weight [28]. The environmental conditions our sample faced related to poverty, such as insufficient resources for reliable and healthy food, and living in unsafe neighborhoods, can work together to constrain and limit individual behavior and ability to control dietary intake.

Another potential reason for the lack of impact on weight gain may be the late timing and short duration of the intervention. The intervention started as late as week 20 of pregnancy. A substantial number of women enrolled in our study had already gained close to or exceeded the IOM weight gain recommendation before the intervention had started (almost 20%). A sensitivity analysis showed that even excluding women who had excessive weight gain at 20 weeks did not change results. Preventing or reducing excess maternal weight before conception is likely the most effective weight management strategy [63, 64].

There are several significant limitations and methodological issues to note, and some are inherent in early intervention development research. While it is always advantageous to have a randomized control group, in alignment with the ORBIT model, the focus at this stage of the research was to tailor an intervention to meet the needs of the specific population, and to examine mechanisms and efficacy of early outcomes, rather than to conduct a large randomized controlled trial. Our study fulfilled many of the goals of the ORBIT model. We developed an intervention that was highly accepted with good fidelity and attendance [43], and found the intervention group improved on stress, depression, and had better glucose tolerance compared to the treatment as usual group, but found no improvement on weight gain, and the presumed links between stress and weight change were not detected.

A limitation in our design was the lack of ability to sensitively test our mediational model. Proximal mechanisms and behavioral risk factors are best measured with frequent samples of behavior. To test how changes in daily affect, stress, and emotion regulation might be impacting eating behavior, and eventually clinical outcomes like weight, more sensitive measurements than pre–post trait measures are necessary. We recommend that future studies utilize ecological momentary assessments or end-of-day diaries to assess mechanisms and changes in behavior in a more granular way.

Although our assignment strategy based on gestational weeks (as well as inability to attend classes based on schedule) resulted in matched groups on sociodemographic measures, the women in the comparison group were enrolled a few weeks later in their pregnancy, which may have influenced results in unknown ways. The later stage of pregnancy could have biased the TAU group to have even fewer women who were glucose intolerant because we ruled out women with gestational diabetes at enrollment, and they were more likely to know at their later stage of pregnancy. Despite this potential bias, we found more women, not fewer, with glucose intolerance in the TAU group. The women had to be able to attend our class at a certain weekly day and time (typically evening). Women who knew they would not be able to attend most of the classes were included in the control group. The primary reasons for not being able to attend were family and work commitments in the evening or geographic distance. We tried to minimize obstacles by providing $25 per session to offset childcare and/or transportation. The women in the intervention group may have had more control over their schedules, leading to selection bias. However, as noted in Table 1, there were no sociodemographic or psychological differences between the two groups at baseline.

Another limitation of the study was the lack of objectively measured repeated outcomes such as physical activity, and insulin sensitivity, and daily measures that are more sensitive than trait measurement. This was a challenging sample to recruit and maintain, given their low income, limited availability, lack of transportation, unexpected life events, network stressors, and high stress, and for some, housing instability. However, we would in future studies prioritize getting repeated weight measures of all enrolled women and, when possible, objective measurements of health behaviors. It is also notable that almost 40% of eligible women did not respond to our invitation (*n* = 146) or declined (*n* = 17) typically because of difficulty and obstacles in their family and work schedules. If the intervention was part of bundled care, such as in Centering Pregnancy, it would have been easier to reach more women.

It is important to examine race/ethnicity separately from socioeconomic factors, which requires a larger sample size and stratification by SES. Even within our all low-income sample, the non-Hispanic whites tended to have higher education and income than the non-whites and Hispanics. By including only low-income women, we reduced structural confounding, but could not wholly eliminate it. Further, our sample size was not sufficient to examine separate effects of race/ethnicity subgroups and SES.

In conclusion, we found preliminary evidence of several important benefits of a short-term mindfulness intervention for low-income, ethnic-minority pregnant women with overweight or obesity. Given the demonstrated benefits to maternal mental and metabolic health, we believe that this intervention has substantial promise to impact maternal and infant outcomes. The ORBIT model provides the framework for next steps—such as further optimization of intervention, dose and duration, refinement of and more sophisticated measurement protocols of mechanisms, and then testing in a larger randomized clinical trial for efficacy. Efforts to continue to develop this intervention and those like it are desperately needed, especially since pregnancy is a highly influential period for the long-term trajectories of both maternal and offspring health.

## Footnotes

1 Per HHS guidelines, In the Bay Area, $18,000 was the 100% poverty level in 2010 for a 3-person household. We used < 500% of the federal poverty level for a household of 3 ($91,550), which was our median household size in this sample (https://aspe.hhs.gov/2010-hhs-poverty-guidelines) as the upper cut-off, which was close to the median income for San Francisco (https://www.deptofnumbers.com/income/california/san-francisco/). As noted, most of our families were well below this ($25,000 annual income on average).
